# Supplementary material for: Caregiver Burden among Caregivers of Patients with Mental Illness: A Systematic Review and Meta-Analysis
Source: Healthcare (Basel). 2022 Nov 30;10(12):2423. doi: 10.3390/healthcare10122423 (PMC9777672; doi:10.3390/healthcare10122423)
Supplement: Supplementary file 1 [file healthcare-10-02423-s001.zip › healthcare-1995796-supplementary.pdf]

Table S1. Strengthening the Reporting of Observational Studies in Epidemiology (STROBE) of Included Studies.

**Item No†**	**Kohn-Wood & Wilson (2005) [59]***	**Koutra et al. (2016) [67]**	**Magana et al. (2007) [60]***	**Maridal et al. (2021) [51]***	**Okewole et al. (2016) [41]***	**Penteado et al. (2020) [46]**	**Rady et al. (2021) [66]***	**Dos Santos et al. (2020) [48]**	**Udoh et al. (2021) [18]***	**Wong et al. (2012) [58]**	**Abayomi et al. (2015) [42]***	**Andrade et al. (2021) [47]**	**Baykal et al. (2019) [54]**
**1(a)**	0	0	0	0	0	0	0	0	0	0	0	1	0
**1(b)**	1	1	1	1	1	1	1	1	1	1	1	1	1
**2**	1	1	1	1	1	1	1	1	1	1	1	1	1
**3**	1	1	1	1	0	1	0	0	1	1	1	1	1
**4**	0	1	0	0	0	1	1	1	1	1	0	1	0
**5**	1	1	1	1	1	1	1	1	1	1	1	1	1
**6(a)**	1	1	1	1	1	1	1	1	1	1	1	1	1
**7**	1	1	1	1	1	1	1	1	1	1	1	1	1
**8***	1	1	1	1	1	1	1	1	1	1	1	1	1
**9**	0	0	0	0	0	0	0	0	0	0	0	0	0
**10**	0	1	0	0	0	0	0	1	0	0	0	0	0
**11**	0	1	0	0	0	1	1	1	1	1	1	1	1
**12 (a)**	1	1	1	1	1	1	1	1	1	1	1	1	1
**12(b)**	1	1	1	1	1	1	1	1	1	1	1	1	1
**12(c)**	0	0	1	0	0	0	0	1	0	0	1	0	0
**12(d)**	0	0	0	0	0	0	0	1	1	1	1	1	0
**12(e)**	0	0	0	1	0	0	0	0	0	0	1	0	0
**13(a)**	0	1	0	0	0	0	0	1	0	0	0	0	0
**13(b)**	0	1	0	0	0	0	0	1	0	0	0	0	0
**13(c)**	0	0	0	0	0	0	0	0	0	0	0	0	0
**14(a)**	1	1	1	1	1	1	1	1	1	1	1	1	1
**14(b)**	0	0	0	0	0	0	0	0	0	0	1	0	0
**15**	1	1	1	1	1	1	1	1	1	1	1	1	1
**16(a)**	0	1	0	0	0	0	0	1	0	1	1	0	1
**16(b)**	0	1	1	1	1	0	0	1	1	1	1	1	0
**16(c)**	0	0	0	0	0	0	0	0	0	0	0	0	0
**17**	0	0	1	1	0	0	0	1	1	0	0	0	0
**18**	1	1	1	1	1	1	1	1	1	1	1	1	1
**19**	1	1	1	1	1	1	1	1	1	1	1	1	1
**20**	1	1	1	1	1	1	1	1	1	1	1	1	1
**21**	1	1	1	0	1	0	1	0	1	1	0	0	0
**22**	0	0	1	1	0	1	0	0	1	1	0	1	0
**Total Score**	10	14	12	12	10	11	10	15	14	14	14	13	10
**Risk of Bias**	**High**	**Low**	**High**	**High**	**High**	**High**	**High**	**Low**	**Low**	**Low**	**Low**	**High**	**High**

Note. *Studies included in meta-analysis. †Names of item 1a to 22 of the STROBE checklist is available from this link: file:///E:/Download/STROBE-checklist-v4-cross-sectional.pdf

Table S1. Strengthening the Reporting of Observational Studies in Epidemiology (STROBE) of Included Studies. (cont.)

Item No	Caqueo-Urizar & Gutie´rrez-Maldonado (2006) [65]*	Chen et al. (2010) [61]*	Cicek et al. (2013) [55]*	Dada et al. (2011) [43]*	Dalky et al. (2017) [72]*	Lorenzo et al. (2021) [70]*	Dols et al. (2018) [78]*	Fan & Chen (2011) [62]	Gonçalves-Pereira et al. (2013) [75]	Gresswell et al. (2018) [69]*	Grover et al. (2015) [68]	Hadrys et al. (2011) [74]	Hanzawa et al. (2008) [71]*
**1(a)**	0	0	0	1	0	0	0	0	0	0	0	0	0
**1(b)**	1	1	1	1	1	1	1	1	1	1	1	1	1
**2**	1	1	1	1	1	1	1	1	1	1	1	1	1
**3**	1	1	0	0	1	1	1	1	1	0	0	1	0
**4**	0	0	0	1	1	1	1	1	1	1	1	0	0
**5**	1	1	1	1	1	1	1	1	1	1	1	1	1
**6(a)**	1	1	1	1	1	1	1	1	1	1	1	1	1
**7**	1	1	1	1	1	1	1	1	1	1	1	1	1
**8**	1	1	1	1	1	1	1	1	1	1	1	1	1
**9**	0	0	1	0	1	1	0	0	0	0	0	0	0
**10**	0	0	0	0	0	1	1	1	1	0	0	0	0
**11**	1	1	1	1	1	1	1	1	1	1	1	1	1
**12 (a)**	1	1	1	1	1	1	1	1	1	1	1	1	1
**12(b)**	1	1	1	1	1	1	1	1	1	1	1	0	0
**12(c)**	0	0	0	0	1	0	1	0	0	0	0	0	0
**12(d)**	0	0	0	0	1	1	0	0	0	0	0	0	0
**12(e)**	0	0	0	0	0	0	0	0	0	0	0	0	0
**13(a)**	0	0	0	0	1	1	1	1	1	0	0	1	0
**13(b)**	0	0	0	0	1	0	1	1	0	0	0	1	0
**13(c)**	0	0	0	0	0	0	0	0	0	0	0	0	0
**14(a)**	1	1	1	1	1	1	1	1	1	1	1	1	1
**14(b)**	0	0	0	0	0	0	1	1	0	0	0	0	0
**15**	1	1	1	1	1	1	1	1	1	1	1	1	1
**16(a)**	0	0	0	0	0	1	0	1	1	0	0	1	0
**16(b)**	0	0	0	0	0	1	0	1	0	0	0	0	1
**16(c)**	0	0	0	0	0	0	0	0	0	0	0	0	0
**17**	0	0	0	0	0	0	0	0	0	0	0	0	0
**18**	1	1	1	1	1	1	1	1	1	1	1	1	1
**19**	0	1	1	1	1	1	1	1	1	1	1	1	0
**20**	1	1	1	1	1	1	1	1	1	1	1	1	1
**21**	0	0	1	1	1	0	1	0	1	0	1	0	1
**22**	0	0	0	0	1	1	0	0	1	1	0	0	1
**Total Score**	**8**	**10**	**10**	**11**	**15**	**15**	**15**	**15**	**14**	**10**	**10**	**11**	**10**
**Risk of Bias**	**High**	**High**	**High**	**High**	**Low**	**Low**	**Low**	**Low**	**Low**	**High**	**High**	**High**	**High**

Note. *Studies included in meta-analysis. †Names of item 1a to 22 of the STROBE checklist is available from this link: file:///E:/Download/STROBE-checklist-v4-cross-sectional.pdf

Table S1. Strengthening the Reporting of Observational Studies in Epidemiology (STROBE) of Included Studies. (cont.)

Item No	Inogbo et al. (2017) [44]	Sharma et al. (2018) [52]*	Tan et al. (2012) [76]*	Fekih-Romdhane at al. (2020) [63]*	Igberase et al. (2012) [45]*	Kizilirmak et al. (2016) [56]*	Mishra et al. (2017) [52]*	Pedroso et al. (2019) [49]*	Scazufca et al. (2002) [50]*	Li et al. (2007) [64]*	Zahid & Ohaeri (2010) [73]*	Chien et al. (2007) [57]*	Reinares et al. (2006) [77]
1(a)	0	1	1	1	0	0	0	1	0	0	0	1	1
1(b)	1	1	1	1	1	1	1	1	1	1	1	1	1
2	1	1	1	1	1	1	1	1	1	1	1	1	1
3	0	0	1	0	1	0	0	1	1	0	0	0	0
4	1	1	1	1	1	1	1	1	1	1	0	1	0
5	1	1	1	1	1	1	1	1	1	1	1	1	1
6(a)	1	1	1	1	1	1	1	1	1	1	1	1	1
7	1	1	1	1	1	1	1	1	1	1	1	1	1
8	1	1	1	1	1	1	1	1	1	1	1	1	1
9	0	0	0	0	0	0	0	0	1	0	0	1	0
10	0	0	0	1	0	1	0	0	0	0	1	1	0
11	1	1	1	1	1	1	1	1	1	1	1	1	1
12 (a)	1	1	1	1	1	1	1	1	1	1	1	1	1
12(b)	0	0	0	0	0	0	1	0	0	0	0	0	0
12(c)	0	0	0	1	0	0	0	0	0	0	0	0	0
12(d)	0	1	1	1	1	1	0	1	1	1	1	1	1
12(e)	0	0	0	0	0	0	0	0	0	0	0	0	0
13(a)	0	0	0	1	0	1	0	1	1	1	1	1	1
13(b)	0	0	0	1	0	1	0	0	0	0	1	1	0
13(c)	0	0	0	0	0	0	0	0	0	0	0	0	0
14(a)	1	1	1	1	1	1	1	1	1	1	1	1	1
14(b)	0	0	0	1	0	1	0	0	0	0	0	1	0
15	1	1	1	1	1	1	0	1	1	1	1	1	1
16(a)	1	0	0	0	0	0	0	0	1	0	1	1	1
16(b)	0	1	1	1	1	1	1	0	1	1	1	1	1
16(c)	0	0	0	0	0	0	0	0	0	0	0	0	0
17	0	0	0	0	0	0	0	0	0	0	0	0	0
18	1	1	1	1	1	1	1	1	1	1	1	1	1
19	1	0	1	1	0	1	1	1	1	1	1	1	1
20	1	1	1	1	1	1	1	1	1	1	1	1	1
21	0	0	0	1	0	1	0	1	1	1	1	1	1
22	0	0	0	0	0	1	0	1	1	0	1	1	1
**Total Score**	**10**	**10**	**12**	**15**	**10**	**15**	**10**	**13**	**15**	**12**	**14**	**16**	**13**
**Risk of Bias**	**High**	**High**	**High**	**Low**	**High**	**Low**	**High**	**High**	**Low**	**High**	**Low**	**Low**	**High**

Note. *Studies included in meta-analysis. †Names of item 1a to 22 of the STROBE checklist is available from this link: file:///E:/Download/STROBE-checklist-v4-cross-sectional.pdf

Table S2. Characteristics of Included Studies.

No	Author	Care Recipients	Country	Study Design	Study Setting	Care Recipient Age	Total Sample Caregiver	Mean Age of Caregivers	Relationship with Patients	Measures for Burden	No. of Items	Function Of Measure	Dimension/Subscale	Score Range/Scoring
1	Kohn-Wood & Wilson (2005) [59]*	Psychotic disorder	USA	Did not mention	Community	17- to 76-year-olds	49	58.0	Biological /conjugal related family member	Feetham Family Functioning Scale	21	To measure perceived satisfaction with aspects of familial functioning.	Did Not Mention	Did Not Mention
2	Koutra et al. (2016) [67]	Psychotic disorder	Greece	Cross-sectional	Hospital	17- to 40-year-olds	100	54.6	Parents	The Family Burden Scale	23	To measure perceived burden of the presence of a psychiatric disorder in a family member	Impact on daily activities, social life, aggressiveness, impact on health, economic burden.	Three-point scale, incorporating the dimension of frequency,ranging from "often" (0), "sometimes" (1), "never" (2).
3	Magana et al. (2007) [60]*	Schizophrenia	USA	Cross-sectional	Clinic	17 years old and above	85	55.1	Primary family caregiver	Zarit Burden Scale	29	To measure the problems perceived by the caregiver with her or his ill family members.	Health, psychological well being, finances, social life,and the relationshipbetween the caregiver and the ill family member.	0-20 (little or no burden)<br>21-40 (mild to moderate burden)<br>41 to 60 (moderate to severe burden)<br>60 and above (severe burden)
4	Maridal et al. (2021) [51]*	Neurodevelopmental disorder (NDD)	Nepal	Did Not Mention	Community	2- to 12-year-olds	63	31.5	Primary caregiver	Self-Designed Questionnaire	7	To measure the impact of the disabled child on central aspects of the caregiver's life and situation.	Household economy, caregiver's physical health, workload, social life, marital relationship, dreams and expectations for their future, and effects on other siblings.	0 (no negative impact), 1 (negative impact), or 2 (very negative impact).
5	Okewole et al. (2016) [51]*	Neuropsychiatric disorders	Nigeria	Did Not Mention	Clinic	Children and adolescents	155	41.5	Did not mention	Zarit Burden Interview	22	To determine the level of burden.	Psychological, financial, social, health, and relationship with the patient being cared for.	0-20 (little or no burden)<br>21-40 (mild to moderate burden)<br>41 to 60 (moderate to severe burden)<br>60 and above (severe burden)
6	Penteado et al. (2020) [46]	Down syndrome (DS)	Brazil	Cross-sectional and exploratory study	Online	Adults and older adults	90	Did not mention	Close relatives or professional caregiver	Self-Designed Questionnaire	22	To determine the level of burden.	Did Not Mention	Did Not Mention
7	Rady et al. (2021) [66]*	No specific mental illness	Eygpt	Cross-sectional, comparative, observational study	Hospital	Adults	70	46.1	Family members	Zarit Burden Interview	22	To determine the level of burden.	Psychological, financial, social, health, and relationship with the patient being cared for.	0-20 (little or no burden)<br>21-40 (mild to moderate burden)<br>41 to 60 (moderate to

													severe burden) 60 and above (severe burden)
8	Dos Santos et al. (2020) [48]	Minor psychiatric disorders	Brazil	Cross-sectional	Community	Adults	537	51.1	Did not mention	Zarit Burden Interview	22	To assess and classify the burden.	Psychological, financial, social, health, and relationship with the patient being cared for.	0-20 (little or no burden) 21-40 (mild to moderate burden) 41 to 60 (moderate to severe burden) 60 and above (severe burden)
9	Udoh et al. (2021) [18]*	No specific mental illness	Nigeria	Descriptive based survey	Hospital	Adults	415	Did not mentio n	Family members	Zarit Burden Interview	22	To find out problems perceived by caregiver of his or her ill family member.	Psychological, financial, social, health, and relationship with the patient being cared for.	0-20 (little or no burden) 21-40 (mild to moderate burden) 41 to 60 (moderate to severe burden) 60 and above (severe burden)
1 0	Wong et al. (2012) [58]*	No specific mental illness	Hong Kong	Cross-sectional survey design	Community	Adults	276	Did not mentio n	Did not mention	Perceived Chronic Strains Scale	14	The severity of perceived strains experienced by caregivers in their day-today care of their mentally ill relatives	Managing drug compliance and follow-up, difficulty managing bizarre and disturbing behavior, handling negative symptoms of persons, and social costs associated with the constant care of people with mental illness.	Not stressful at all' (1) to 'Very stressful' (4)
1 1	Abayomi et al. (2015) [42]*	No specific mental illness	Nigeria	Cross-sectional	Clinic	60 years old and above	128	42.1	Primary caregiver	Zarit Burden Interview	22	To determine the level of caregiver burden.	Burden in the relationship, emotional well-being, social life, finances, and loss of control over one's life.	Total score equal or more than the median (high burden) Total scores below the median (low burden)
1 2	Andrade et al. (2021) [47]	No specific mental illness	Brazil	Cross-sectional	Community	Adults	61	46.4	Family members	Family Burden Interview Schedule	70	To determine the level of caregiver burden.	Financial burden, disruption of routine family activities, disruption of family leisure, disruption of family interaction, effect on physical health of others, and effect on mental health of others.	The items are scored on 3-point scales (no burden=0, moderate burden=1 and severe burden=2). Higher scores indicate the severity of the magnitude of the burden.
1 3	Baykal et al. (2019) [54]	Autistic spectrum disorder (ASD)	Turkey	Cross-sectional	Clinic	Childre n	70	Did not mentio n	Primary caregiver	Zarit Caregiver Burden Scale	19	To examine the effect of caregiving on the life of the individual concerned.	Psychological tension and impaired private life, irritability and restrictedness, impaired social relations, economic burden and dependence.	0-20 (little or no burden) 21-40 (mild to moderate burden) 41 to 60 (moderate to severe burden) 60 and above (severe burden)

14	Caqueo-Urízar et al. (2006) [65]*	Schizophrenia	Chile	Cross-sectional	Community	Adults	41	54.2	Family members	Zarit Caregiver Burden Scale	22	To determine the level of burden.	Burden, rejection and incompetence.	0-20 (little or no burden) 21-40 (mild to moderate burden) 41 to 60 (moderate to severe burden) 60 and above (severe burden)
15	Chen et al. (2010) [61]*	Major depressive disorder	Taiwan	Cross-sectional	Hospital	Older adults	34	55.1	Spouse or children	The Caregiver Burden Inventory (Cbi)	24	To determine the level of burden.	Time dependence, developmental, physical, social, and emotional burdens.	Five-point likert scale that ranged from 0 (not at all descriptive) to 4 (very descriptive)
16	Cicek et al. (2013) [55]*	Obsessive-compulsive Disorder	Turkey	Case-control cross-sectional	Clinic	Adults	47	40.2	First-degree relatives	Zarit Burden Interview	22	To assess the perceived burden in the caregivers of subjects with psychiatric disorders.	Psychological, financial, social, health, and relationship with the patient being cared for.	0-20 (little or no burden) 21-40 (mild to moderate burden) 41 to 60 (moderate to severe burden) 60 and above (severe burden)
17	Dada et al. (2011) [43]*	No specific mental illness	Nigeria	Cross-sectional	Hospital	Children and adolescents	155	41.5	Family caregivers	Zarit Burden Interview	22	To determine the level of burden.	Psychological, financial, social, health, and relationship with the patient being cared for.	0-20 (little or no burden) 21-40 (mild to moderate burden) 41 to 60 (moderate to severe burden) 60 and above (severe burden)
18	Dalky et al. (2017) [72]*	No specific mental illness	Northern Jordan	Cross-sectional Correlation	Clinic	Adults	266	44 37.0	Family members	Caregiver Strain Index	13	To measure of burden to identify family caregivers with potential caregiving concerns.	Employment, financial, physical, social and time.	Items of the CSI are scored as yes (1) or no (0) and then summed. Positive responses (answered yes) to seven or more items on the index indicate an elevated level of strain and that caregivers need further evaluation.
19	Lorenzo et al. (2021) [70]*	Schizophrenia	Italy	Cross-sectional	Community	Adults	60	56.5	Did not mention	Zarit Burden Interview	22	To measure caregiver burden.	Psychological, financial, social, health, and relationship with the patient being cared for.	< 21 not present or mild burden 22–40 mild to moderate burden 41–60 moderate to severe burden > 60 severe burden.
20	Dols et al. (2018) [78]*	No specific mental illness	The Netherlands	Cross-sectional	Community	Older adults	47	58.3	Primary caregiver	Self-Perceived Pressure By Informal Care Scale (SPPIC)	9	To measure burden on the caregiver.	Did Not Mention	Individual items were subsequently dichotomized into (1) some degree of pressure by being a caregiver (scores 4–2), and (0) no pressure by being a

														caregiver (scores 1 and 0)
21	Fan et al. (2011) [62]	No specific mental illness	Taiwan	Cross-sectional	Clinic	Adults	90	Did not mention	Did not mention	Family Problems Questionnaire	34	To access caregiver's burden.	Objective burden, subjective burden, support received, the relative’s positive attitude toward the patient and the relative’s criticism of the patient’s behaviour.	Four-level scale, from ‘always’ to ‘never’.
22	Gonçalves-Pereira et al. (2013) [75]	Schizophrenia	Portugal	Cross-sectional	Community	Adults	108	60.1	Primary caregiver	Involvement Evaluation Questionnaire	81	To provide extensive international comparisons regarding the levels of family burden.	Worrying, tension, supervision and urging.	The items are scored on a 5-point Likert scale (never, sometimes, regularly, often and always). Higher score indicates higher burden.
23	Gresswell. et al. (2018) [69]*	Bipolar disorder	Ireland	Cross-sectional	Community	Adults	53	64.5	Did not mention	Zarit Burden Interview	22	To measure caregiver burden.	Psychological, financial, social, health, and relationship with the patient being cared for.	0-20 (little or no burden) 21-40 (mild to moderate burden) 41 to 60 (moderate to severe burden) 60 and above (severe burden)
24	Grover et al. (2015) [68]	Bipolar affective disorder and schizophrenia	Northern India	Cross-sectional	Hospital	Adults	100	50.3	Did not mention	The Family Burden Interview Schedule	24	To measure caregiver burden.	Financial burden, disruption of routine family activities, disruption of family leisure, disruption of family interaction, effect on physical health of others, and effect on mental health of others.	The items are scored on 3-point scales (no burden=0, moderate burden=1 and severe burden=2). Higher scores indicate the severity of the magnitude of the burden.
25	Hadrys et al. (2011) [74]	No specific mental illness	Poland	Cross-sectional	Hospital	Adults	141	Did not mention	Did not mention	Involvement Evaluation Questionnaire	27	To assess the level of care the caregiver had to provide to the relative in the last 4 weeks, personal problems between them in that period, as well as the caregiver’s various worries.	Worrying, tension, supervision and urging.	The items are scored on a 5-point Likert scale (never, sometimes, regularly, often and always). Higher score indicates higher burden.
26	Hanzawa et al. (2008) [71]*	Schizophrenia	Japan	Cross-sectional	Community	10- to 69-year-olds	57	Did not mention	Family members	Zarit Caregiver Burden Interview	8	To measure caregiver burden.	Psychological, financial, social, health, and relationship with the patient being cared for.	0-20 (little or no burden) 21-40 (mild to moderate burden) 41 to 60 (moderate to severe burden) 60 and above (severe burden)
27	Inogbo et al. (2017) [44]	Schizophrenia	Nigeria	Did not mention	Hospital	Adults	255	45.1		Zarit Burden Interview	22	To provide caregivers' report on their experiences of caregiving.	Psychological, financial, social, health, and	0-20 (little or no burden) 21-40 (mild to

									First degree relatives				relationship with the patient being cared for.	moderate burden) 41 to 60 (moderate to severe burden) 60 and above (severe burden)
28	Sharma et al. (2018) [52]*	Bipolar affective disorder and schizophrenia	Nepal	Cross-sectional	Hospital	Did not mention	100	43.0	Did not mention	Caregiver Strain Index	13	To measure of burden to identify family caregivers with potential caregiving concerns.	Social, psychological, physical, time, financial and employment.	It is scored on a scale of 0-2, where 2-stands for "Yes", 0 for "No" and 1 for "sometimes". Higher score indicates higher caregiver stress.
29	Tan et al. (2012) [76]*	Schizophrenia	Singapore	Did not mention	Community	Did not mention	150	45.6	Did not mention	The Burden Assessment Scale	19	To measure burden objectively and subjectively.	Financial problems, limitations on personal activity, household disruption social interactions, feelings, attitudes and emotions.	Four-point Likert scale items, with 1 denoting 'not at all' to 4 denoting 'a lot', '
30	Fekih-Romdhane at al. (2020) [63]*	Schizophrenia and Bipolar Disorders	Africa	Cross-sectional	Hospital	60 years old and above	52	48.4	Family members	Zarit Burden Interview	12	To provide caregivers' report on their experiences of caregiving.	Psychological, financial, social, health, and relationship with the patient being cared for.	0-20 (little or no burden) 21-40 (mild to moderate burden) 41 to 60 (moderate to severe burden) 60 and above (severe burden)
31	Igberase et al. (2012) [45]*	Schizophrenia	Nigeria	Cross-sectional	Hospital	Did not mention	200	Female-53.8 Male-50.7	Primary caregivers	Burden Questionnaire	13	To access burden among relatives of individuals with psychotic illness.	Financial loss, impairment of work efficiency, disruption of routine family activities, disruption of family interactions and physical health, what caregivers think about the illness and the general feeling of difficulty experienced in caring for the patient.	The summary burden score for each domain of objective burden is derived by adding up the scores on the relevant items ranging from 1 to 4.
32	Kizilirmak et al. (2016) [56]*	No specific mental illness	Turkey	Cross-sectional	Hospital	Did not mention	243	42.2	Family members	Burden Assessment Scale	19	To assess the care burden of caregivers of patients with severe mental disorders.	Feelings of distress, financial strain and feelings of stigma.	The scale is a 19 item that is scored on 4-point Likert style scale as follows: not at all = 1, very little = 2, some = 3, a lot = 4. The total score of the scale is the arithmetical total of all items. Higher scores indicate that the caregiver burden is high.
33	Mishra et al. (2017) [52]*	Schizophrenia	Nepal	Cross-sectional	Hospital	Did not mention	36	31.2	Family members	Burden Assessme	40	To assess both objective and subjective burden experienced by the	Subjective burden and objective burden.	Items rated on a 3-point scale, marked 1-3. The responses

										nt Schedule		primary care givers of chronic mentally ill patients.		would be "not at all, to some extent or very much".
34	Pedroso et al. (2019) [49]*	No specific mental illness	Brazil	Cross-sectional	Hospital	Did not mention	112	49.0	Family members	Zarit Burden Interview	12	To provide caregivers' report on their experiences of caregiving.	Psychological, financial, social, health, and relationship with the patient being cared for.	0-20 (little or no burden)<br>21-40 (mild to moderate burden)<br>41 to 60 (moderate to severe burden)<br>60 and above (severe burden)
35	Scazufca et al. (2002) [50]*	depression	Brazil	Cross-sectional	Clinic	60 years old and above	82	55.4	Did not mention	Zarit Burden Interview	22	To provide caregivers' report on their experiences of caregiving.	Psychological, financial, social, health, and relationship with the patient being cared for.	0-20 (little or no burden)<br>21-40 (mild to moderate burden)<br>41 to 60 (moderate to severe burden)<br>60 and above (severe burden)
36	Li et al. (2007) [64]*	Schizophrenia	China	Descriptive correlation study	Hospital	Did not mention	96	Did not mention	Family members	Caregiver Burden Scale	14	To measure the family caregiver's objective burden, subjective stress burden, and subjective demand burden.	objective burden, subjective stress burden, and subjective demand burden.	The objective burden means > 23, the subjective burden means > 15, and the subjective stress burden means > 13.5 are viewed as high.
37	Zahid & Ohaeri (2010) [73]*	Schizophrenia	Kuwait	Cross-sectional	Hospital	Less than 65 years old	121	36.8	Principal family caregivers	Involvement Evaluation Questionnaire	81	To provide extensive international comparisons regarding the levels of family burden.	Worrying, tension, supervision and urging.	The items are scored on a 5-point Likert scale (never, sometimes, regularly, often and always). Higher score indicates higher burden.
38	Chien et al. (2007) [57]*	Schizophrenia	Hong Kong	Cross-sectional	Clinic	Did not mention	203	45.2	Family members	The Family Burden Interview Schedule (FBIS)	25	To measure caregiver burden.	Financial burden, disruption of routine family activities, disruption of family leisure, disruption of family interaction, effect on physical health of others, and effect on mental health of others.	The items are scored on 3-point scales (no burden=0, moderate burden=1 and severe burden=2). Higher scores indicate the severity of the magnitude of the burden.
39	Reinares et al. (2006) [77]	Bipolar Disorders	Spain	Cross-sectional	Hospital	18- to 65-year-olds	86	49.1	Family members	Social Behaviour Assessment Schedule	Did not mention	To assess the subjective burden.	Distress associated with the patient's behaviour, the patient's role performance and the adverse effects on others both inside and outside the household	0=no distress;<br>1=moderate distress;<br>2=severe distress).

Note. *Studies included in meta-analysis (n=26).
